# Integrated *de novo* transcriptome of *Culex pipiens* mosquito larvae as a resource for genetic control strategies

**DOI:** 10.1038/s41597-024-03285-1

**Published:** 2024-05-09

**Authors:** Valentina Mastrantonio, Pietro Libro, Jessica Di Martino, Michele Matera, Romeo Bellini, Tiziana Castrignanò, Sandra Urbanelli, Daniele Porretta

**Affiliations:** 1 Department of Environmental Biology, Sapienza University of Rome, 00185 Rome, Italy; 2 Department of Ecological and Biological Sciences, Tuscia University, Largo dell’Università snc, 01100 Viterbo, Italy; 3 Envu, 2022 ES Deutschland GmbH, Germany, Monheim, Germany; 4 Department of Vector Biology, Liverpool School of Tropical Medicine, Pembroke Place, Liverpool, L3 5QA United Kingdom; 5 Centro Agricoltura Ambiente “G. Nicoli”, Via Sant’Agata 835, 40014 Crevalcore, Italy

**Keywords:** Ecology, Computational biology and bioinformatics

## Abstract

We present a *de novo* transcriptome of the mosquito vector *Culex pipiens*, assembled by sequences of susceptible and insecticide resistant larvae. The high quality of the assembly was confirmed by TransRate and BUSCO. A mapping percentage until 94.8% was obtained by aligning contigs to Nr, SwissProt, and TrEMBL, with 27,281 sequences that simultaneously mapped on the three databases. A total of 14,966 ORFs were also functionally annotated by using the eggNOG database. Among them, we identified ORF sequences of the main gene families involved in insecticide resistance. Therefore, this resource stands as a valuable reference for further studies of differential gene expression as well as to identify genes of interest for genetic-based control tools.

## Background & Summary

Insecticide resistance is a serious problem in the control of vectors and vector-borne diseases (VBDs)^1^. In the last decades, many efforts have been addressed to reveal the mechanisms underlying insecticide resistant phenotypes and develop new tools alternative to chemical compounds. Genetically-based tools (e.g., RNA interference, CRISPR-Cas9) have been identified as a cutting-edge alternative to manage insecticide resistance because they enhance our ability to detect, functionally validate, and inhibit putative resistance genes and/or mutations^2,3^. Likewise, these tools are proving effective in suppressing critical biological functions, inducing sex ratio distortion, and driving the spreading of desired genes within populations^3–6^. However, these technologies have a primary constraint to be effectively applied: the need for available target sequences. Increasing high-quality -omics resources is therefore mandatory, especially for non-model species.

Transcriptomes have recently been considered valuable genomic resources^7–12^. Contrary to genomic data revealing what genes are present within cells, transcriptomes provide evidence about what genes are expressed under certain conditions. By comparing transcriptome data of different species, life stages, tissues, or conditions, we can thus elucidate the molecular pathways underlying specific phenotypes and identify candidate genes for traits of interest^13–17^. Nonetheless, this possibility to explore the multiple facets of transcriptomes mainly relies on high-quality references for comparison.

Here, we furnish a *de novo* assembled transcriptome of the mosquito *Culex pipiens* (ecotype *pipiens*), the primary vector of several pathogens such as West Nile virus, Japanese encephalitis, and lymphatic filariasis across the temperate Northern Hemisphere^18,19^. This species belongs to the *Culex pipiens* complex, that also include *Cx. quinquefasciatus*, *Cx. pipiens pallens*, *Cx. australicus* and *Cx. globocoxitus*^20^ and it is commonly found in urban and natural habitats. Due to its vector competence, *Cx. pipiens* has always been a priority in chemicals control applications. Recently, concern about this species has also increased because genetic resistance to the insecticide diflubenzuron (DFB), the principal larvicide used to control *Cx. pipiens*, has been found in populations across its geographic range^21–25^. Genetic analyses showed that DFB resistance was associated with a mechanism already observed in other relevant arthropod pests^2,26,27^, where point mutations in the chitin-synthase I gene (chs1) cause aminoacidic substitutions from wildtype Isoleucine into mutated Leucine, Methionine or Phenylalanine (i.e., I1043L; I1043M; I1043F)^2,20,22,28,29^. A deep analysis of the homozygous I1043M-resistant strain of *Cx. pipiens* established from field populations also revealed that resistant mosquitoes had an increased cuticle thickness and constitutively up-regulate the chs1 gene^30^.

Since including transcripts expressed under different conditions improves the broad use of the transcriptomic references, the *de novo* transcriptome of *Cx. pipiens* presented in this paper has been assembled by including in the dataset susceptible and I1043M-resistant 4-^th^ instar larvae, which represent the target of DFB applications^31^. The bioinformatic analysis was conducted following field-best practices, employing specialized tools for quality control, sequence alignment, and transcriptomic assembly. In particular, we adopted widely recognized methodologies to ensure an accurate and comprehensive representation of the *Cx. pipiens* transcriptome. Subsequent annotation was performed using standard transcriptomic annotation procedures, ensuring a thorough understanding of the functionality of the identified transcripts. Furthermore, ORF sequences belonging to detoxifying gene families associated with insecticide resistance, such as ABC transporters, cytochrome P450, glutathione-S-transferases, UDP-glucosyltransferase, cuticular proteins and heat shock proteins were identified within the *Cx. pipiens* transcriptome.

Our resource will be a valuable reference for future studies about insecticide resistance and future comparative transcriptomic studies about mosquito biology. We cannot exclude that other mechanisms beyond target site mutation in the *chs* 1 gene could be associated with DFB resistance in *Cx. pipiens*. As shown by several studies, the analysis of constitutive differentially expressed genes in susceptible and resistant individuals is a main approach to identifying genes associated with insecticide resistance^32–35^. Likewise, the analysis of differentially expressed genes under insecticide exposure could allow us to identify a larger pool of transcripts and reveal further genes associated with insecticide resistance. From a technological perspective, the transcriptome of *Cx. pipiens* will be also useful to identify new genes of interest for genetic control tools.

## Methods

### Mosquitoes

Mosquitoes used to generate the dataset were obtained from colonies established from field populations of *Cx. pipiens*. Immature mosquitoes were collected in the sites of Parma (Lat. 44,768382; Long. 10,319429) and Forlì (Lat. 44,21091241; Long. 12,05524495) to establish the DFB susceptible and resistant colonies, respectively. In each site, at least five breeding sites were sampled to have a representative sample of the population^30^. The susceptible colony was homozygous for the wild-type allele I1043, while the resistant colony was homozygous for the resistant allele I1043M^30^. Mosquitoes from 5^th^ generation of both colonies were used for the current study.

Larval stages of both colonies were maintained in plastic trays containing 2 L of spring water and daily fed with fish food (0.85 mg/larva) (Tetra® Goldfish Granules). Adults were maintained in 45 × 45 × 45 cm Bugdorm insect-rearing cages (Watkins & Doncaster, UK) and daily fed with 10% sucrose solution and water ad libitum. Artificial blood meal was also provided to females to mature and lay eggs (Hemotek Ltd, UK). Immature and adult stages were all reared in a thermostatic chamber with constant conditions of temperature, relative humidity (RH), and photoperiod (L:D) (i.e., T = 26 ± 1 °C; RH = 70%; L:D = 16:8 hours light:dark). Two thousand L_1_ larvae of each strain were put in plastic trays filled with 2 L of spring water, and the trays were set up in triplicate. In each tray, the larvae were maintained as described above, and at L_4_, a pool of 10 larvae was collected and immediately stored in RNA later buffer (Thermo fisher Scientific, Ravenna, Italy) at −80 °C until RNA extraction^36–38^.

### RNA isolation, library preparation and sequencing

Pooled RNA was extracted from 6 pools of whole-body 4^th^-instar larvae of *Cx. pipiens* (i.e, three pools for each colony). Extraction was performed using whole-body tissues to include a larger pool of RNAs in the *de novo* transcriptome assembly of *Culex pipiens*. RNA extraction was performed using the NucleoSpin RNA Plus XS kit (Macherey-Nagel, AG), following the manufacturer’s instructions. A step including a DNase treatment was also performed during RNA extraction. After RNA isolation, the quality and integrity of RNAs were checked by QubitTM fluorimetry and the 5200 Fragment Analyzer (Agilent Technologies, USA). Libraries preparation and sequencing were performed by the Polo GGB (Polo d’Innovazione di Genomica, Genetica e Biologia), Perugia, Italy (http://www.pologgb.com/) using a NextSeq Illumina® platform. The libraries were prepared in accordance with the QIAseqTM Stranded mRNA Selected Kit Handbook for Illumina Paired-End Indexed Sequencing. Briefly, oligo-dT probes were covalently attached to the surface of the magnetically charged Pure mRNA Beads, and the bound RNA was then washed and eluted to provide a highly enriched pool of mRNA. Then the enriched mRNA was fragmentated by heat according to input RNA quality and approximate insert size. After fragmentation, enriched mRNA was converted to cDNA using an RNase H in combination with random primers. Once the synthesis of the first strand finished, the second-strain synthesis was performed using 5′ phosphorylated random primers. Samples underwent adapter ligation, and then the indexed libraries were amplified, purified, and validated. After a normalization step, libraries were pooled in equal volumes for sequencing. The Illumina NextSeq 550 sequencing system was used through the Illumina chemistry V2,2x75bp paired end run. On average 43.7 million reads for each library were obtained and they are available at the NCBI Sequence Read Archive (project ID PRJEB47420).

### Pre-assembly processing stage

**Fig. 1:**
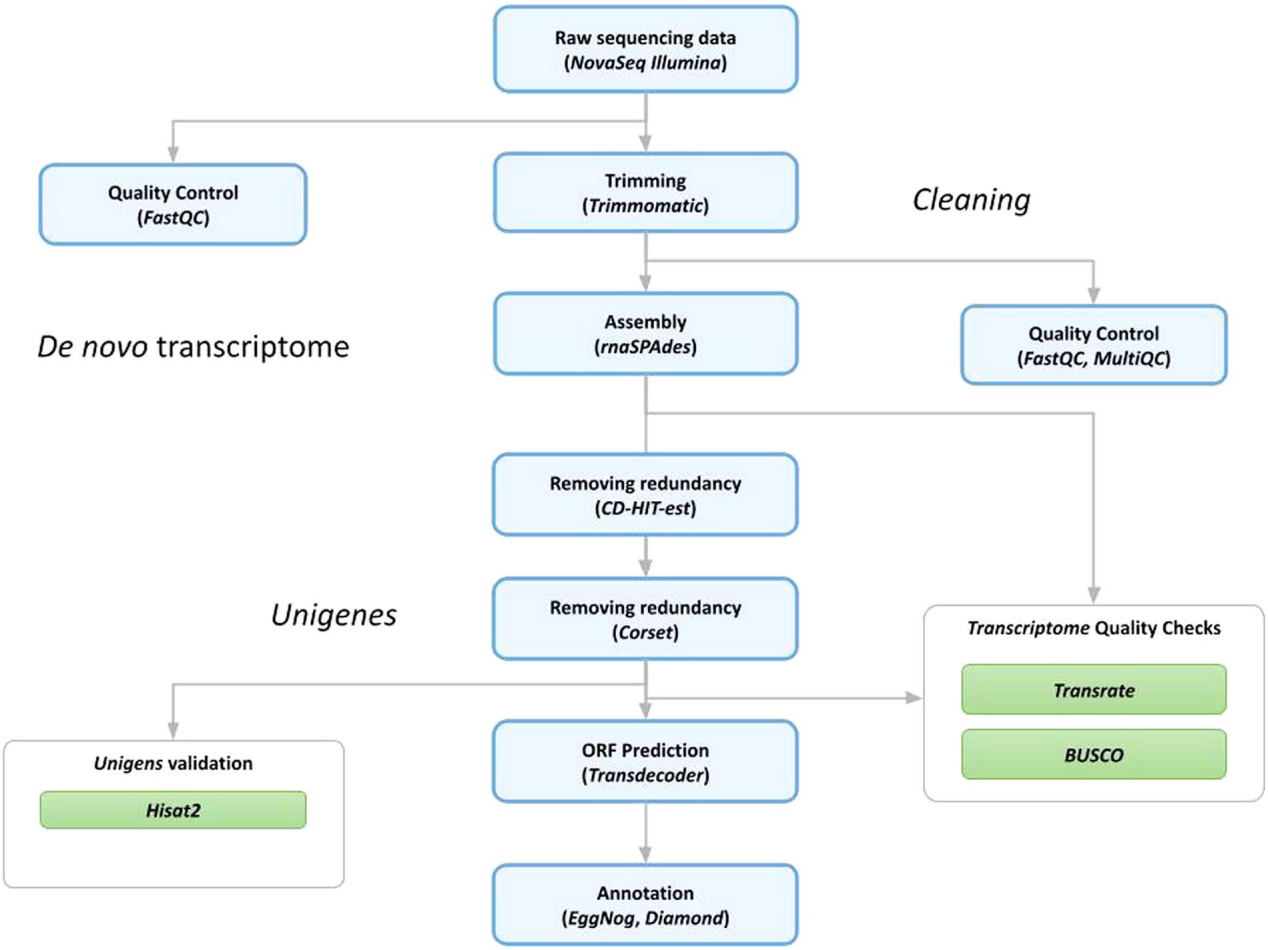
Workflow of the bioinformatic pipeline for the *de novo* transcriptome assembly of *Culex pipiens*, starting from raw data and leading to annotated scripts. Each step is sequentially numbered.

The bioinformatics analyses described below were conducted using high-performance computing systems through the ELIXIR-IT HPC@CINECA’s call^39–43^. The workflow of the bioinformatics pipelines, adapted from two previous studies^44,45^, is illustrated in Fig. 1. A total of 437,233,732 pairs of reads was produced through Illumina sequencing. All of them underwent a cleaning analysis process to prepare data for assembly. The FastQC 0.11.5 tool (http://www.bioinformatics.bbsrc.ac.uk/projects/fastqc) was used to assess the quality of the initial reads, which allowed to estimate the quality profiles of the RNAseq samples. Quality estimates with FastQC were performed on both raw and trimmed data. In order to eliminate adapter sequences and low-quality bases, an initial analysis of the raw reads was conducted using Trimmomatic, v.0.39^46^ (setting the option SLIDINGWINDOW: 4: 15, MINLEN: 36, and HEADCROP: 13). All reads that were unpaired were excluded from further analysis. Executing the trimming process, a total of 391,907,976 clean reads were retained for constructing the *de novo* transcriptome assembly (which equates to approximately 90% of the original raw reads, as shown in detail in Table 1). To provide a summary quality assessment metrics view for processed data quality, results were combined across all samples and compiled into a report using the MultiQC21 v.1.9 software tool^47^ (see steps 2–4 of Fig. 1). The analysis results were deposited on figshare (see Imagefile 2 on Table 2).

**Table 1:** Summary of the 6 libraries deposited in the European Nucleotide Archive (ENA, BioProject: PRJEB47420)^67^, in terms of number of raw and trimmed reads per sample.

Run ID	Phenotypes	RNA concentration (ng/ul)	RQN	Raw sequences	Filtered sequences (% of the total reads)
ERR10360688	DFB-Resistant	66	7,6	75,657,372	65,583,016 (87%)
ERR10360689	DFB-Resistant	60	9,6	83,210,660	76,111,468 (91%)
ERR10360692	DFB-Resistant	66	7,9	61,782,376	56,336,296 (91%)
ERR10360695	Susceptible	70	10	68,850,032	62,820,900 (91%)
ERR10360696	Susceptible	70	7,2	76,598,300	68,146,132 (89%)
ERR10360699	Susceptible	80	6,2	71,134,992	62,910,164 (88%)

Information about RNA concentration and integrity (RNA Quality Number, RQN) were also shown. For RQN, higher values indicate a higher quality total RNA sample (reported on a scale of 1 to 10).

**Table 2:** Overview of produced data files and their access on figshare^68^.

Label	Name of data	File types	Data repository (URL)
Image file 1	Per sequence quality scores (made with MultiQC)	PDF file (.pdf)	10.6084/m9.figshare.23014226
Image file 2	Mean quality scores (made with MultiQC)	PDF file (.pdf)	10.6084/m9.figshare.23014184
Data file 1	rnaSPAdes RNA-seq *de novo* transcriptome assembly	Fasta file (.fasta)	10.6084/m9.figshare.22512946
Data file 2	CD-HIT-est output (Unigenes)	Fasta file (.fasta)	10.6084/m9.figshare.22514845
Data file 3	Corset output	Fasta file (.fasta)	10.6084/m9.figshare.22515256
Data file 4	Open reading frames (ORFs) prediction	Fasta file (.cds)	10.6084/m9.figshare.22515262
Data file 5	Blastx vs Nr (Functional annotation from non-redundant (Nr) NCBI)	Text file (.tsv)	10.6084/m9.figshare.22561345
Data file 6	Blastx vs SwissProt (Functional annotation from Swiss-Prot)	Text file (.tsv)	10.6084/m9.figshare.22561423
Data file 7	Blastx vs TrEMBL (Functional annotation from TrEMBL UniProt)	Text file (.tsv)	10.6084/m9.figshare.22561450
Data file 11	Eggnog output	Text file (.tsv)	10.6084/m9.figshare.23581590
Data file 12	Proteins of *Culex pipiens pallens*	Fasta file (.faa)	10.6084/m9.figshare.23581704
Data file 13	Proteins of *Culex quinquefasciatus*	Fasta file (.faa)	10.6084/m9.figshare.23581728
Data file 14	Proteins of *Aedes aegypti*	Fasta file (.faa)	10.6084/m9.figshare.23581731
Data file 15	Proteins of *Anopheles gambiae*	Fasta file (.faa)	10.6084/m9.figshare.23581743
Data file 16	Statistics comparison (OrthoFinder output)	Text file (.txt)	10.6084/m9.figshare.23581752
Data file 17	Orthogroups (OrthoFinder output)	Text file (.tsv)	10.6084/m9.figshare.23581770
PDF file 1	List of commands of the applied bioinformatics pipeline	PDF file (.pdf)	10.6084/m9.figshare.24559084
Data file 18	Sequences of predicted ORFs associated to gene families involved in insecticide resistance	Fasta file (.fasta)	10.6084/m9.figshare.25355656

### *De novo* transcriptome assembly and quality assessment

Since the species under study lacks a specific annotated reference genome^48^, we proceeded with the *de novo* assembly of the transcriptome. We used the assembler rnaSPAdes^49^, a tool for *de novo* transcriptome assembly from RNA-Seq data, available in the SPAdes v.3.14.1 package. Using rnaSPAdes, we generated a total of 50,416 assembled transcripts, with a CG content of 47% and an N50 of 2,409 bp, as presented in Table 3.

**Table 3:** Statistics on rnaSPAdes, CD-HIT-est and Corset outputs, evaluated with the Transrate assembly validator.

Validation scores	rnaSPAdes	CD-HIT-est	Corset
**Basic parameters**			
Total transcripts	50416	41054	32252
N50	2409	2254	2208
GC content (%)	47	47	47
**Transrate v.1.0.3**			
Transrate Assembly Score	0.0828	0.1904	0.1902
Transrate Optimal Score	0.1056	0.2041	0.2070
Transrate Optimal Cutoff	0.1868	0.0682	0.0682
Good contigs	36776	38451	29969
p good contigs	0.73	0.94	0.93

In order to ensure the elimination of assembly redundancies, two filtering steps were executed. The initial step involved the utilization of CD-HIT-est (v. 4.8.1) on the output obtained from rnaSPAdes. The resulting data was then uploaded to figshare for further analysis (see Datafile 2 on Table 2). Subsequently, we employed Corset (v. 1.06)^50^, a tool that has demonstrated its efficacy in a previous study^51^, to generate the final assembly. The Corset output showed an N50 of 2208 bp (Table 3). As a result of removing redundancies in an efficient way (two steps of removal), the final assembly contained about 63.94% of the original transcripts.

**Fig. 2:**
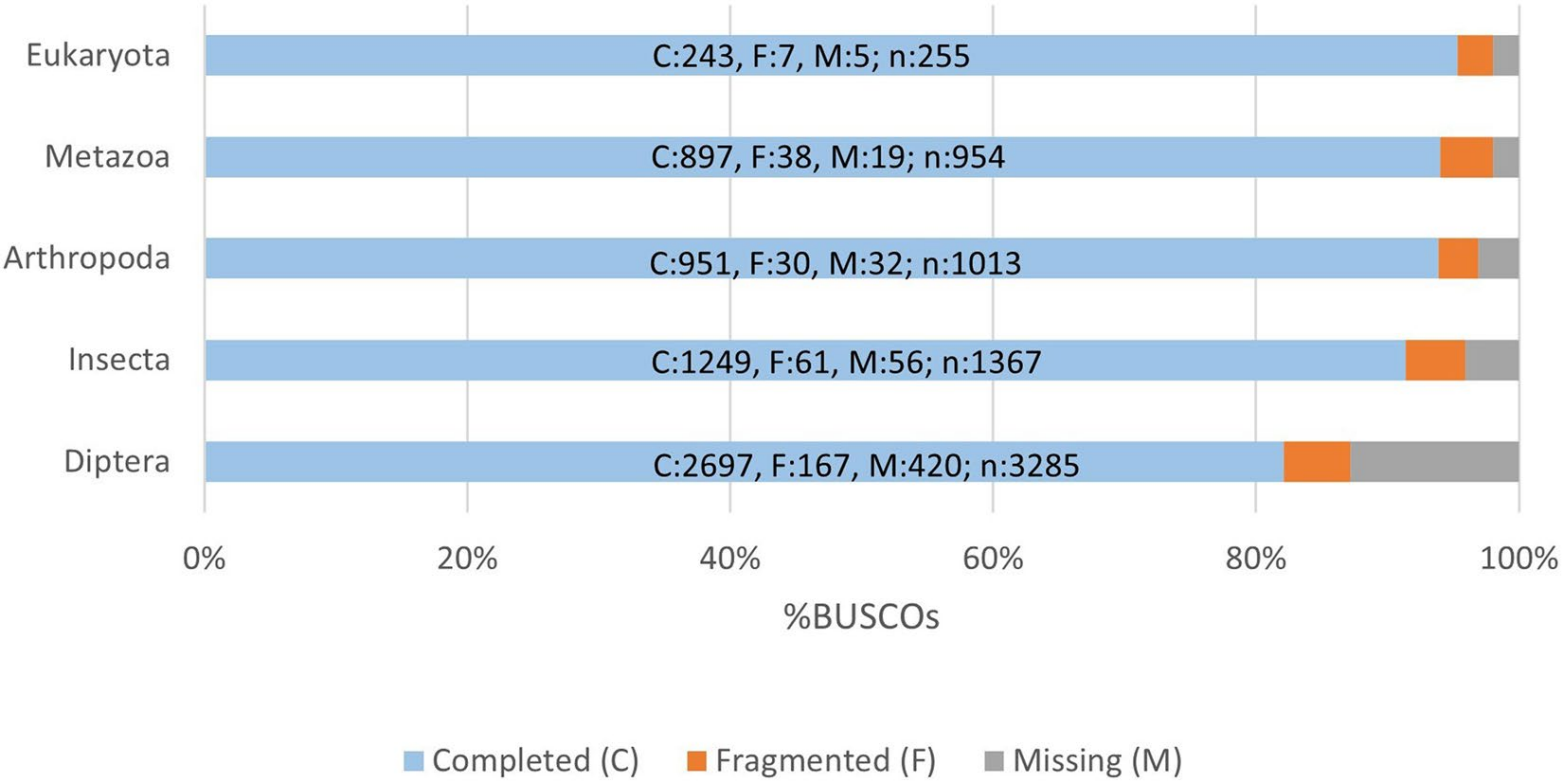
BUSCO assessment results.

The evaluation of the assembly results involved two validation phases: one conducted after the assembly process to assess the initial assembly, and another performed after removing redundancies to evaluate the quality of the final non-redundant assembly. To accomplish this, two distinct software tools were employed, namely TransRate^52^ (v. 1.0.3) and BUSCO (Benchmarking Universal Single-Copy Orthologs)^53^ (v. 5.4.4). These tools generate a range of metrics that serve as indicators for identifying potential errors in the assembly process and provide evidence regarding the transcriptome’s quality. BUSCO offers a quantitative measure of transcriptome completeness and quality by comparing gene content against evolutionarily informed expectations derived from databases of near-universal and highly conserved protein orthologs. In this study, BUSCO analysis was performed using five orthologous gene databases: Arthopoda, Metazoa, Eukaryota, Insecta and Diptera. The degree of transcriptome completeness, as assessed by BUSCO, is presented in Table 4, while Fig. 2 illustrates the distribution of completed, fragmented, and missing genes from the four databases. Furthermore, the quality assessment of the Corset output involved the use of HISAT2 (v. 2.1.0)^54^ to map trimmed reads back to the reference transcriptome (unigenes). The results of all the validation phases can be found in Table 2 and are extensively discussed in the subsequent “Technical Validation” section.

**Table 4:** The BUSCO (vs. 5) validation, though the gVolante web server to five databases.

Busco Category	Eukaryota	Metazoa	Arthropoda	Insecta	Diptera
Complete BUSCOs (C)	243 (95.3%)	897 (94.03%)	951 (93.8%)	1249 (91,37%)	2697 (82,10%)
Complete and single-copy BUSCOs (S)	237 (92.9%)	855 (89.6%)	880 (86.9%)	1182 (86,5%)	2541 (77,5%)
Complete and duplicated BUSCOs (D)	6 (2.4%)	42 (4.4%)	71 (7.0%)	67 (4,9%)	151 (4,6%)
Fragmented BUSCOs (F)	7 (2.7%)	38 (4.0%)	30 (3.0%)	61 (4,5%)	167 (5,1%)
Missing BUSCOs (M)	5 (2.0%)	19 (2.0%)	32 (3.1%)	56 (4,1%)	420 (12,8%)
Total BUSCO groups searched	255	954	1013	1367	3285

### Generation of the full-length transcriptomes

Following the initial validation and evaluation phase, which involved the use of TransRate and BUSCO, the output of the assembly procedure served as the input for CD-HIT-est program^55^. CD-HIT-est is a hierarchical clustering tool utilized to eliminate redundant transcripts and fragmented assemblies, ensuring the generation of unique genes during the *de novo* assembly process. The default parameters of CD-HIT-est were employed, setting a 95% similarity threshold. To further refine the final transcriptome dataset, a subsequent hierarchical clustering phase was performed using Corset. Subsequently, the Corset output, after the validation phase with Hisat2, was subjected to analysis with TransDecoder^56,57^ (v. 5.7.0), a widely recognized tool for identifying long open reading frames (ORFs) within assembled transcripts. The default parameters of TransDecoder were used, enabling ORF prediction on both strands of assembled transcripts regardless of the sequenced library. Additionally, TransDecoder ranked the ORFs based on their completeness and assessed if the 5′ end was incomplete by examining the presence of any length of amino acid codons upstream of a start codon (M) without a stop codon. The “Longest ORF” rule was adopted, selecting the translation start site with the highest 5 AUG (relative to the inframe stop codon).

### Transcriptome annotation

The contigs obtained from the assembly were subjected to ORFs prediction performed with Transdecoder (vs. 5.5.0). For the predicted ORFS of the *de novo* assembly, we employed a variety of annotations. They were aligned against the Nr, SwissProt, and TrEMBL databases using the DIAMOND algorithm to extract the most relevant annotations.DIAMOND^58^ is an open-source algorithm that offers a significant improvement in speed compared to BLASTX, making it highly suitable for short reads. With a comparable level of sensitivity, DIAMOND employs a double indexing approach to exhaustively determine all meaningful alignments for a given query. Traditional sequence comparison programs, such as BLASTX, typically follow a seed-and-extend paradigm. This two-phase approach involves searching for matches of seed sequences (short segments of the query sequence) in the reference database, followed by an extension phase aimed at computing a complete alignment. To configure DIAMOND, we employed the following parameter settings: DIAMOND-fast DIAMOND BLASTX-t 48 -k 250 -min-score 40 for a faster analysis, and DIAMOND-sensitive: DIAMOND BLASTX -t 48 -k 250 -sensitive -min-score 40 for a more sensitive analysis. These settings ensured efficient and accurate annotation of the assembly.

For each database, Nr, SwissProt, and TrEMBL, we selected the best annotation, resulting in the creation of an annotation matrix. The predicted ORFs s were analysed using BLASTX, the tool performing nucleotide-to-protein sequence searches. The use of BLASTX against Nr, TrEMBL, and SwissProt yielded the following results: 40576 (92.7%), 41507 (94.8%), and 27289 (62.3%) contigs, respectively. These results are in line with raw reads mapping observed in the *de-novo* transcriptomes annotation of other species^11,59,60^. Detailed information on the resulting datasets can be found in Table 5. Furthermore, an overview of the data files and datasets generated in this study, along with relevant details on the data repository and access numbers, is summarised in Table 2.

**Table 5:** Summary of homology annotation hits performed with BLASTX and on three different databases: Nr, SwissProt and TrEMBL.

Database	Number of BLASTX results
Nr	40576 (92.7%)
TrEMBL	41507 (94.8%)
SwissProt	27289 (62.3%)

**Fig. 3:**
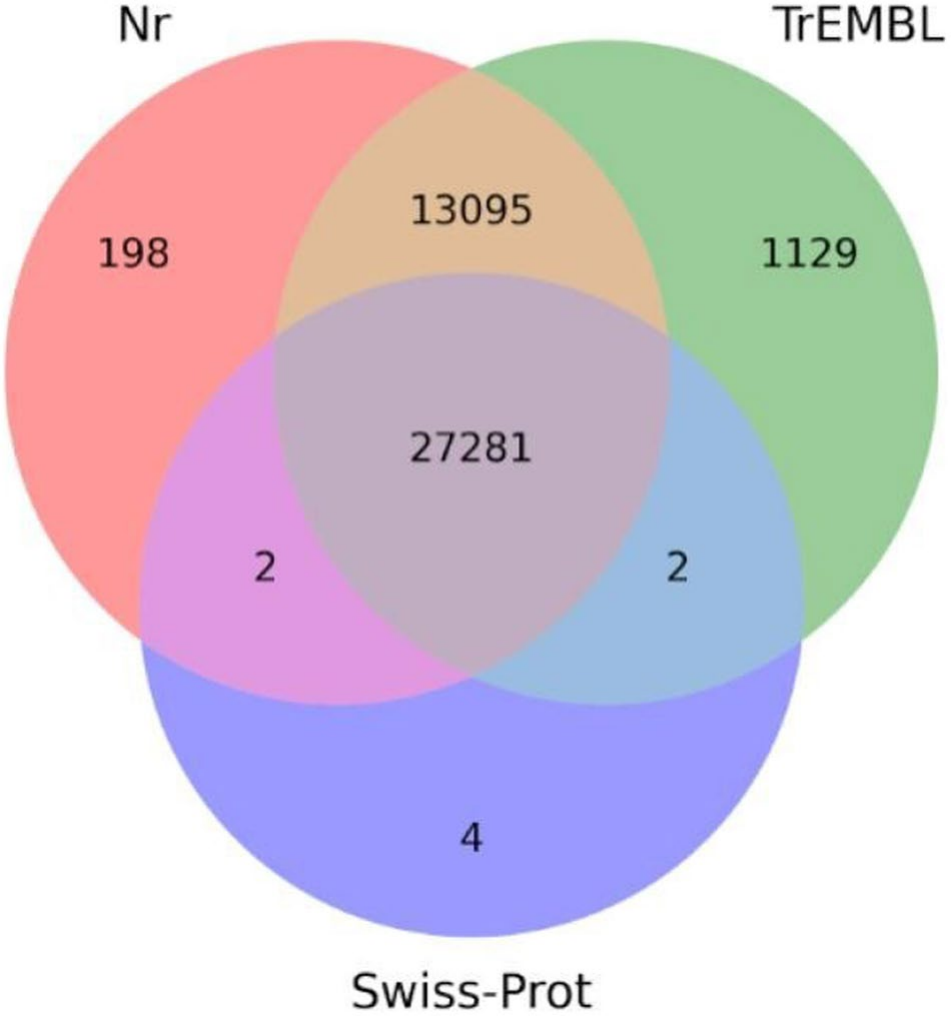
Venn diagrams for the number of contigs annotated with DIAMOND (BLASTX) against the three databases: Nr, SwissProt, TrEMBL. The number of unique and shared contigs for each database is shown.

**Fig. 4:**
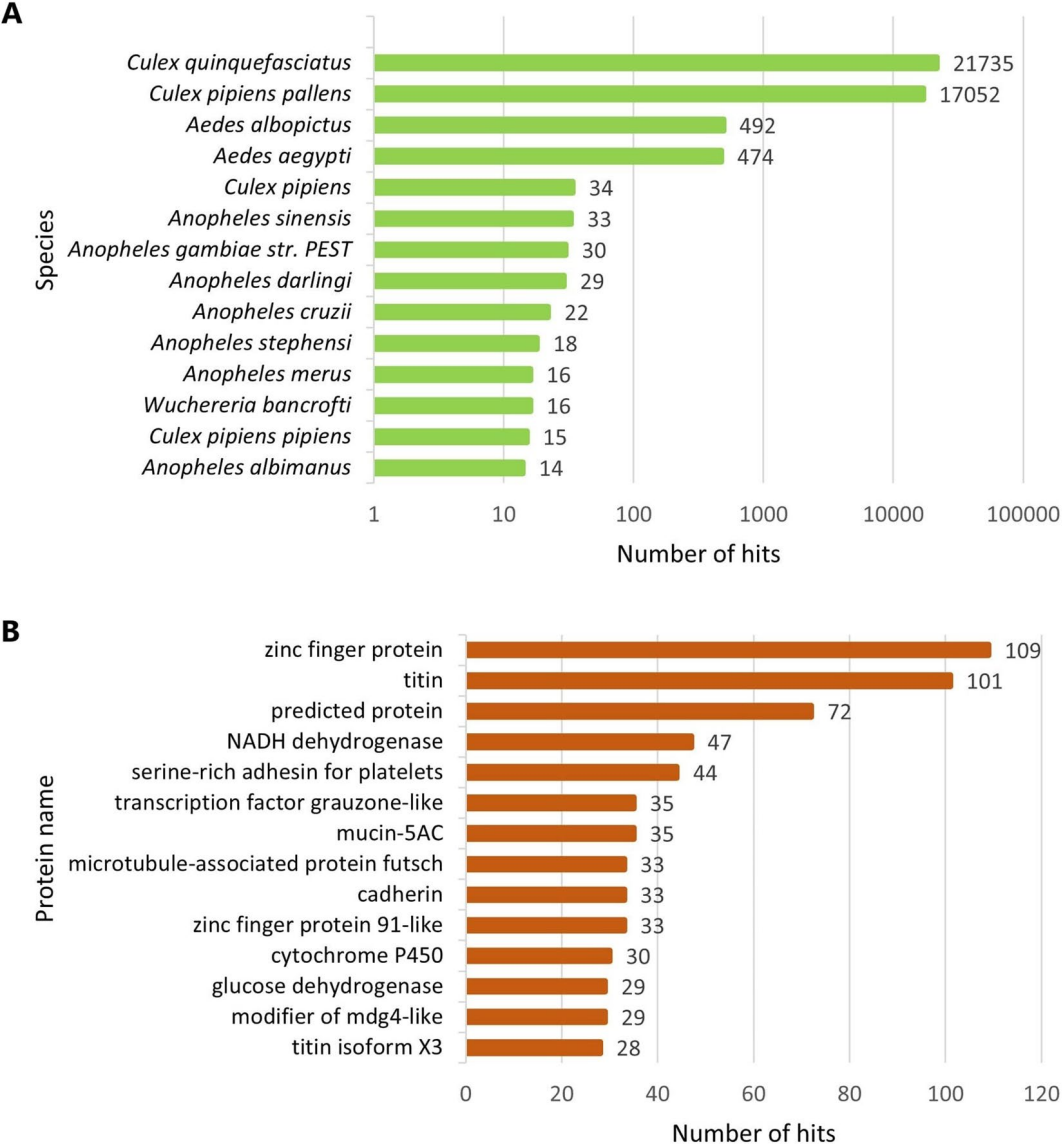
Most represented species and gene product hits obtained with BLASTX by mapping the transcripts against the reference database Nr. Panel (**A**) shows the top 10 best species (A) and Panel (**B**) shows the protein hits present in the reference database.

**Fig. 5:**
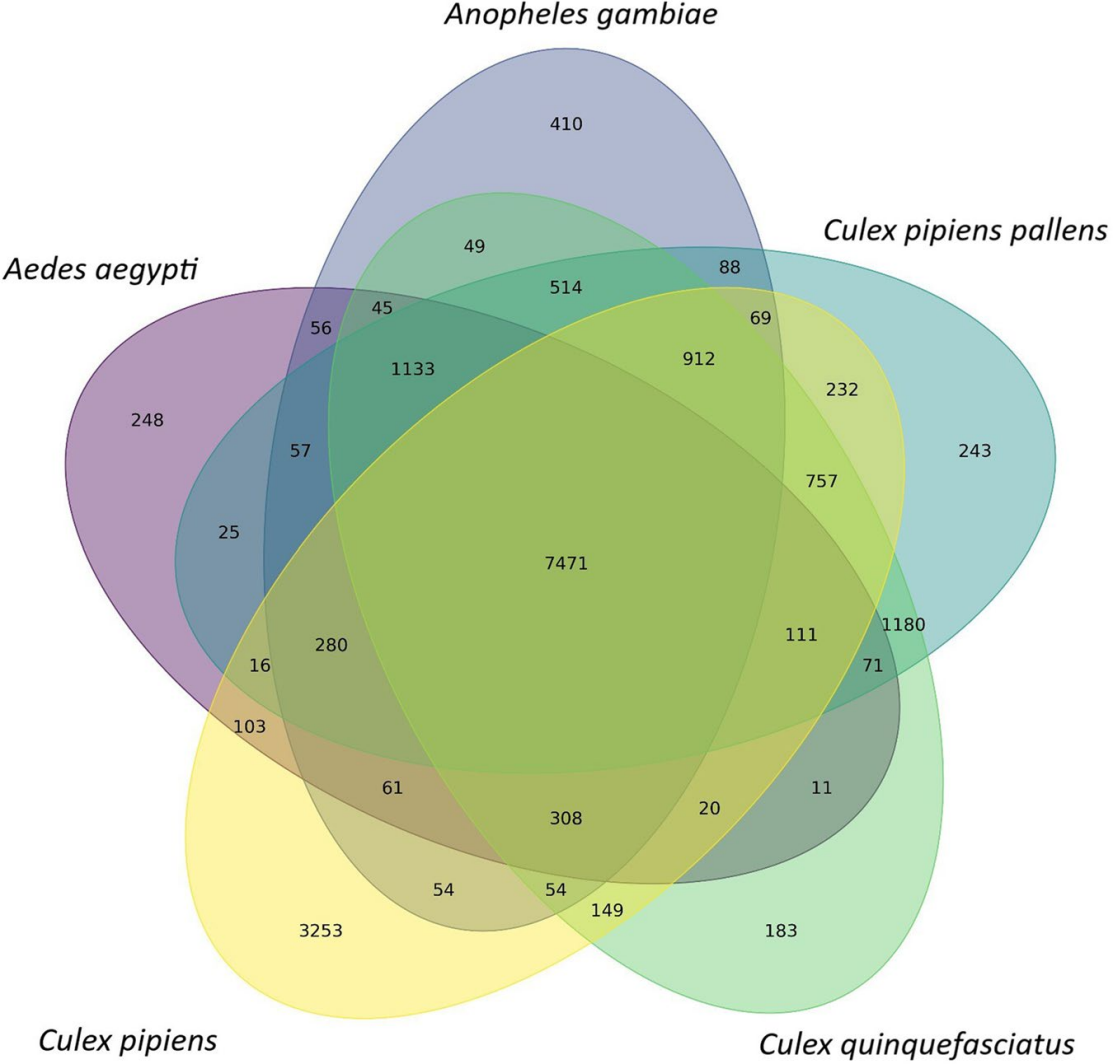
Venn diagram representing the number of species-specific and overlapping protein orthogroups between the five transcriptome assemblies. The number of orthogroups were identified with OrthoFinder.

The results of the BLASTX annotation yielded a total of 27,281 sequences that were simultaneously mapped to the Nr, SwissProt, and TrEMBL databases. Venn diagrams illustrating the overlap of annotations in different databases are presented in Fig. 3, showcasing the redundancy of annotations for both DIAMOND BLASTX. Additionally, Figs. 4, 5 display the top ten most represented species and gene product hits obtained from BLASTX by aligning the transcripts with the reference database Nr. Furthermore, the ten most represented species and the ten hits of the gene product obtained with BLASTX by mapping the transcripts against the reference database Nr are shown in Fig. 4.

The total number of predicted ORFs obtained from the transcriptome assembly were also mapped onto another database of functional annotations: eggNOG (Evolutionary genealogy of genes: Non-supervised Orthologous Groups)^61^. The eggNOG database incorporates various taxonomic levels of orthologous groups (OGs) of proteins with functional annotations, using an algorithm that exploits previous orthologous group (COG) methodologies. Of the 43,793 total predicted ORFs obtained in our analyses, 14,966 (or 34.2%) were annotated in the eggNOG database. For details, see Datafile 11 in Table 2.

### Selection of ORF sequences belonging to gene families related to insecticide resistance

Regulatory changes of genes involved in the oxidation, conjugation and extrusion of chemical compounds is a main mechanism associated with insecticide resistance^62^. By using an ad-hoc parsing script, we searched within the annotation file of predicted ORFs for gene families known be involved in insecticide resistance. Then, we reported in Table 6 the number of found predicted ORFs in the NR annotation file by this search. Furthermore, in Table 2, we added the link to the FASTA file deposited in figshare (‘Data file 18’), including the nucleotide sequences of predicted ORFs of gene families related to insecticide resistance. Further research comparing mosquitoes exposed to insecticide and under control conditions would be necessary identify additional genes involved in DFB resistance and/or to validate the data presented here.

**Table 6:** Number of ORFs with Nr annotations belonging to gene families of interest known for insecticide resistance^50^.

Detoxifying gene family	Number of ORFs
ATP-binding cassette (ABC) transporters	61
Cytochrome P450	163
Glutathione-S-transferase	16
UDP-glucosyltransferase	22
Cuticular proteins	228
Heat shock proteins	25

### Comparison with other species through the orthologues

The comparison of orthologous genes with those of closely related is a crucial step in validating the quality of a *de novo* transcriptome assembly, as outlined by several evidence in literature^63–65^. Here, therefore we compared ORFs predicted from the *de novo* transcriptome of *Culex pipiens* with all proteins potentially transcribed based on the assembled genomes of *Aedes aegypti, Anopheles gambiae, Culex pipiens pallen*s and *Culex quinquefasciatus*. The identification and orthological grouping of all proteins of the various species was performed with OrthoFinder^66^ (v. 2.5.5). This approach also served to assess the completeness of the assembly on the basis of sequence similarity. OrthoFinder allows the identification of orthogroups, defined as a set of genes that descend from a single gene of the last common ancestor within species groups. A total of 136,663 genes were identified, grouped into 18,163 orthologues. Of these, only 12,764 genes were classified as species-specific and were grouped into 4,337 deduced orthologues. In fact, orthogroup detection showed considerable overlap in sequences in all five groups. Over 40% (7471) of the transcripts identified as putative orthologues were shared between all five species (Fig. 5). Consequently, a relatively low proportion of transcripts were identified as unique to a given assemblage (i.e., “species-specific” or “assembly-specific”); notably only 248 transcripts (1.4%) in *Aedes aegypti*, 410 transcripts (2.3%) in *Anopheles gambiae*, 243 (1.3%) in *Culex pipiens pallens*, 183 (1.0%) in *Culex quinquefasciatus* and 3253 transcripts (17.9%) in *Culex pipiens* were classified as species-specific. Therefore, the marked level of sequence overlap observed between the transcriptomes further validates the completeness and quality of the assemblage presented in this study. In addition to providing inference of the completeness of the assemblage, these results represent the first transcriptome-level comparison of four ecologically important Culicidae species. Interestingly, we found no marked difference in the number of overlapping sequences between the focal species in terms of their phylogenetic distance/proximality and each other (Fig. 5).

## Data Records

All raw data generated in this project have been deposited in the European Nucleotide Archive (ENA, BioProject: PRJEB47420). The *de novo* transcriptome assembly resource is available on figshare (link: Data file 3 in Table 2). The datasets containing all files produced in this transcriptome assembly and annotation pipeline (rnaSPAdes transcriptome assemblies, unigenes and functional annotation files) have also been deposited in the figshare archive (links to the pipeline results are listed in Table 2).

## Technical Validation

### Raw reads quality and validation of the assembly

**Fig. 6:**
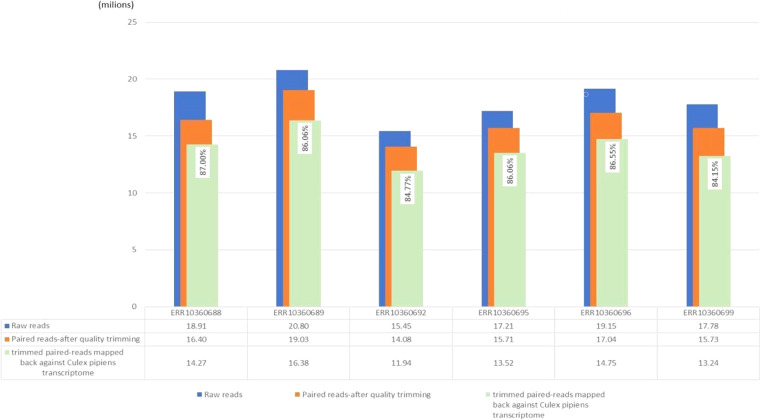
For each sample, the representation of the total paired-reads is shown in blue, the total paired-reads after removal of the adapters and quality trimming is shown in orange, and the mapped trimmed paired-reads compared to the *de novo* assembled transcriptome of *Culex pipiens* is shown in green.

The overall data quality was assessed using FastQC for all samples before and after cutting. Among the FastQC results, the average quality scores at each base position were above 35 (see image file 1 in Table 2). Validation of the transcriptome assembly was performed using two validation tools: BUSCO and TransRate. The results of the validation steps are shown in Table 3. BUSCO analysis was performed on five databases: Arthopoda, Metazoa, Eukaryota, Insecta and Diptera. The details of BUSCO are listed in Table 4 and some of them are represented, in the form of a histogram, in Fig. 2. A further validation evaluation was performed by mapping the clipped reads against the *de novo* assembled transcriptome of *Culex pipiens*. The HISAT2 results showed an even higher percentage of 86% (Fig. 6), confirming the high quality of the assembly. The final transcriptome (unigenes) obtained after CD-HIT-est comprised a total of 41,054 transcripts and an N50 of 2254 bp, with a completeness value of over 80% for the BUSCO evaluation in every database interrogated.

### Quality control of annotation

The transcriptome was functionally annotated by running DIAMOND and eggNOG. The application of DIAMOND for annotation resulted in the identification of 43,793 predicted ORFs (for BlastX analysis) shared between the three databases. EggNOG is a comprehensive orthologous gene database that provides detailed functional information for genes within each orthologous group. The EggNOG database includes a wide range of sequenced genomes from different species, providing a robust evolutionary context for our data analysis. The EggNOG analysis provided valuable insights through COG (Cluster of Orthologous Groups) assignments and KEGG (Kyoto Encyclopedia of Genes and Genomes) annotations.

## Data Availability

All the software programs used in this article (*de novo* transcriptome assembly, pre- and post-assembly steps and transcriptome annotation) are listed with the version in the Methods paragraph. In case of no details on parameters the programs were used with the default settings.
